# Severe Hypokalemic Paralysis Unmasking Renal Tubular Acidosis in a Patient With Sjögren’s Syndrome

**DOI:** 10.7759/cureus.92567

**Published:** 2025-09-17

**Authors:** Harisanth Rajaram, Sherwin Ganegoda, Kesavan Sivanesan, Aishwarya Chitnis, Naim Ahmadouk

**Affiliations:** 1 General Medicine, Royal Berkshire NHS Foundation Trust, Reading, GBR; 2 General Medicine, Oxford University Hospitals NHS Foundation Trust, Oxford, GBR; 3 Emergency Medicine, Royal Papworth Hospital, Cambridge, GBR; 4 Gastroenterology, Frimley Health NHS Foundation Trust, Frimley, GBR; 5 Acute and Renal Medicine, Buckinghamshire Healthcare NHS Trust, Aylesbury, GBR

**Keywords:** autoimmune disease, distal renal tubular acidosis, electrolyte imbalance, hypokalemic periodic paralysis, rheumatology, sjögren’s syndrome

## Abstract

Hypokalemic periodic paralysis (HPP) is a rare but life-threatening complication in patients with Sjögren’s syndrome (SS), often due to distal renal tubular acidosis (RTA) caused by autoimmune-mediated renal damage. We report a case of a woman in her 30s with a history of rheumatoid arthritis and SS who presented with acute, flaccid paralysis secondary to profound hypokalemia from distal RTA, requiring intensive care support. This patient was treated with central potassium and bicarbonate replacement, leading to marked clinical improvement. This report emphasizes the importance of early recognition of HPP as a manifestation of SS-related RTA and underscores why multidisciplinary management and active long-term follow-up are essential to prevent relapse and optimize patient outcomes.

## Introduction

Sjögren’s syndrome (SS) is a chronic autoimmune disorder with a prevalence of up to 2.1% [1]. It is characterized by lymphocytic infiltration of the exocrine glands, most commonly causing sicca symptoms, such as dry eyes and mouth [2]. Renal involvement is one of the more common extraglandular manifestations, with heterogeneous manifestations ranging from electrolyte imbalances to interstitial nephritis, glomerulonephritis, and distal renal tubular acidosis (RTA) [3,4]. RTA can cause profound hypokalemia, a critical electrolyte imbalance that may result in hypokalemic periodic paralysis (HPP) [5]. This case report highlights the importance of recognizing HPP as a manifestation of SS-related RTA, a pertinent differential diagnosis for muscle weakness in a patient with SS.

## Case presentation

A woman in her late 30s presented with a four-week history of generalized muscle weakness, myalgia, dysphagia, and progressive dyspnea. She had a past medical history of seropositive rheumatoid arthritis (RA) and SS overlap, and was not on any disease-modifying anti-rheumatic drugs (DMARDs). She reported chronic sicca symptoms (dry mouth and dry eyes), as well as a six-month history of gradually worsening breathlessness. In the days preceding her admission, she developed profound symmetrical weakness in all four limbs and marked fatigue, prompting presentation to the emergency department for further evaluation.

On examination, she was alert and oriented. Her vital signs were as follows: blood pressure 95/63 mmHg, respiratory rate 19 breaths per minute, heart rate 51 beats per minute, temperature 36.2°C, and oxygen saturation 100% on room air. Cardiovascular, respiratory, and abdominal examinations were unremarkable, except for palpable parotid gland enlargement. Neurological examination revealed flaccid quadriparesis, with Medical Research Council (MRC) grade 1/5 strength in all limbs, globally diminished deep tendon reflexes with preserved sensation and cranial nerve function.

**Figure 1:**
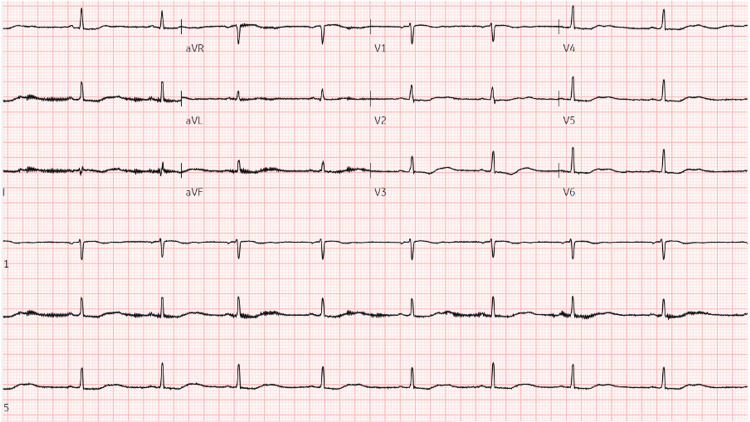
Patient's ECG on admission.

Initial venous blood gas analysis revealed a metabolic acidosis (pH 7.18, bicarbonate 11.5 mmol/L, base excess -15 mmol/L, lactate of 1.2 mmol/L). Serum biochemistry revealed normal sodium (139 mmol/L), severe hypokalemia (potassium 1.7 mmol/L), elevated chloride (116 mmol/L), and elevated creatine kinase (487 IU/L). Immunoglobulin screen revealed a polyclonal hypergammaglobulinemia. The estimated glomerular filtration rate (eGFR) was above 60 mL/min/1.73 m². Urinalysis revealed a spot urine pH of 7, 24-hour urinary potassium excretion of 80 mmol/day, urinary anion gap of 6 mmol/L, and a urine protein creatinine ratio of 66.7 mg/mmol. The calculated serum anion gap was 12 mmol/L, consistent with a hyperchloremic normal anion gap metabolic acidosis (Table 1). An electrocardiogram (ECG) demonstrated sinus bradycardia with a prolonged QTc interval of 590 ms (Figure 1).

**Table 1:** Key laboratory investigations. eGFR: estimated glomerular filtration rate; ESR: erythrocyte sedimentation rate; CRP: C-reactive protein

Serum	Initial values	Reference range
Sodium (mmol/L)	139	136-145
Potassium (mmol/L)	1.7	3.5-5.1
Magnesium (mmol/L)	0.8	0.7-1
Urea (mmol/L)	5	2.5-6.7
Creatinine (μmol/L)	95	50-98
eGFR (mL/min/1.73 m²)	68	>90
Adjusted calcium (mmol/L)	2.24	2.1-2.55
Albumin (g/L)	38	35-50
Chloride (mmol/L)	116	98-107
Creatine kinase (IU/L)	487	29-168
ESR (mm/h)	65	3-12
CRP (mg/L)	3.3	0-5
Blood gas		
pH	7.18	7.35-7.45
Lactate (mmol/L)	1.7	0.5-2.2
HCO_3_ (mmol/L)	11.5	22-26
Base excess (mmol/L)	-15	-2-3
Urine		
pH	7	4.5-8.0
Sodium (mmol/L)	62	-
Potassium (mmol/L)	8	-
Chloride (mmol/L)	64	-
24-hour potassium excretion (mmol/day)	80	<30
Urine protein creatinine ratio (mg/mmol)	66.7	0-30
ECG		
Rate (bpm)	40	60-100
QTc (ms)	590	<460

Imaging included a computed tomography scan of the kidneys, ureters, and bladder, which excluded obstructive uropathy but identified an atrophic right kidney and a bicornuate uterus. An esophago-gastro-duodenoscopy to investigate dysphagia was unremarkable. The elevated creatine kinase prompted an MRI of the femurs, which demonstrated non-specific signal changes in the tensor fascia lata muscles bilaterally, with no radiological evidence of myositis.

Her clinical background, in combination with profound hypokalemia, hyperchloremic normal anion gap metabolic acidosis, increased urinary potassium excretion, and a positive urine anion gap strongly supported a diagnosis of distal (type 1) renal tubular acidosis secondary to SS.

She was promptly admitted to the intensive care unit for urgent management of hypokalemia, where she received central venous catheter (CVC) potassium replacement and peripheral intravenous bicarbonate. After stabilization, she was gradually weaned off CVC potassium replacement and transitioned to high-dose oral therapy with SANDO-K (four tablets, four times daily) and sodium bicarbonate (1 g, four times daily) until discharge.

Prior to discharge, the patient showed significant improvement in her muscle weakness and breathlessness, with normalization of her potassium levels. However, she reported some residual symptoms, including lower limb myalgia, arthralgia exacerbated by cold weather (particularly in the first metatarsophalangeal joints, hand joints, and knees), and ongoing sicca symptoms.

For long-term management, she was started on hydroxychloroquine (200 mg twice daily) with no steroid cover. She had previously tried methotrexate and leflunomide but was unable to tolerate the side effects. She was also prescribed 800 IU of vitamin D daily and advised on the importance of sun protection. With her electrolyte and acid-base balance now stable on oral therapy, she was discharged with active outpatient follow-up by both rheumatology and nephrology.

## Discussion

This case demonstrates a classic example of distal renal tubular acidosis (RTA) in the setting of secondary Sjögren’s syndrome, manifesting as severe hypokalemia and flaccid paralysis. The lack of disease-modifying therapy given this patient's background of autoimmunity may have contributed to the development of the severe electrolyte imbalance seen.

Distal RTA is characterized by impaired hydrogen ion secretion in the α-intercalated cells of the distal nephron, resulting in difficulty acidifying urine despite systemic acidosis. Clinically, this results in a normal anion gap (hyperchloremic) metabolic acidosis, often leading to hypokalemia from the urinary potassium wasting. A positive urine anion gap, a urine pH higher than 5.5, and increased urinary potassium excretion are key diagnostic indicators of distal RTA [6].

In SS, distal RTA originates from lymphocytic infiltration and immune-mediated destruction of the distal nephron, leading to interstitial nephritis and tubular dysfunction [7]. Renal involvement occurs in about 5-15% of people with primary SS, and although distal RTA is the most common renal manifestation, it often remains unnoticed until symptoms become severe. This patient displayed the typical traits of HPP, a rare but recognized outcome of distal RTA [8].

The pathophysiology of HPP occurs due to the role potassium plays in maintaining the resting membrane potential and neuromuscular excitability. A reduction in extracellular potassium hyperpolarizes the resting membrane potential, impairing depolarization and reducing the ability of skeletal muscle fibers to generate action potentials. This results in decreased muscle contractility and, in severe cases, produces flaccid paralysis. Additionally, hypokalemia impairs activity of the sodium-potassium ATPase, further disrupting ion gradients critical for neuromuscular function. Clinically, this imbalance manifests as acute, symmetrical, ascending weakness with preserved sensation, consistent with the flaccid quadriparesis observed in this case [9,10].

The flaccid paralysis in all four limbs, weakened reflexes, and preserved sensation are consistent with muscle weakness induced by severe hypokalemia, likely exacerbated by the underlying autoimmune condition. A review of current literature showed that HPP is a rare manifestation of SS, and timely recognition is critical, as delays in potassium correction can lead to respiratory failure, arrhythmias, and even death [5].

ECG features, such as sinus bradycardia, a prolonged QTc interval, and U waves, are well-recognized signs of severe hypokalemia. The QTc of 590 ms in this patient significantly increased the risk of ventricular arrhythmias, such as torsades de pointes, emphasizing the need for urgent critical care monitoring and treatment [11].

The patient’s laboratory profile, characterized by a normal anion gap metabolic acidosis, elevated urinary potassium loss, and high urine pH, met the criteria for diagnosing distal RTA. The presence of an atrophic kidney was most likely a congenital abnormality as her eGFR remained above 60 mL/min/1.73 m², with normal creatinine levels, indicating adequate overall kidney function [12]. A clinical risk-benefit decision was made not to do a renal biopsy at the time of presentation, given the acuity and supportive biochemical findings of distal RTA.

While distal RTA in SS can typically be managed with potassium and bicarbonate supplements, it is imperative that immunomodulatory therapy is given to help prevent recurrences by controlling the underlying autoimmune activity. Hydroxychloroquine, commonly used in treating SS and RA, was appropriately started in this patient, considering her joint pain, fatigue, and autoimmune indications. While no formal protocols for managing RTA in SS exist, case series suggest that approximately one-third of patients respond with supportive therapy alone, while others need corticosteroids or immunosuppressive agents [13].

## Conclusions

This case contributes to the small but growing body of literature on SS-related RTA and HPP, emphasizing the need for clinicians to recognize and manage these rare complications. Multidisciplinary care and close monitoring are critical to managing symptoms, preventing relapse, and addressing comorbidities. Importantly, treatment should be tailored to the degree of renal involvement, patient's autoimmune profile, and their risk of relapse. In certain cases, supportive management may be sufficient, whereas in others, immunomodulation may be required for systemic control. Future studies are needed to help develop evidence-based guidelines on electrolyte management and the role of immunosuppressive therapy in preventing recurrent HPP in SS-related RTA.
